# Hybridization of multi-objective evolutionary algorithms and fuzzy control for automated construction, tuning, and analysis of neuronal models

**DOI:** 10.1186/1471-2202-14-S1-P369

**Published:** 2013-07-08

**Authors:** Parth Patel, Myles Johnson-Gray, Emlyne Forren, Atish Malik, Tomasz G Smolinski

**Affiliations:** 1 Department of Computer and Information Sciences, Delaware State University, Dover, DE 19901, USA

## 

As of late, automated methods for generation and tuning of neuronal models have been gaining popularity (*e.g*. [1]). Several such techniques, including brute-force parameter search space exploration (*e.g.*[2]) or particle swarm optimization (*e.g.*[3]), have been successfully applied in this area; however, evolutionary/genetic algorithms seem to be the tool of choice for an increasing number of computational neuroscientists (*e.g.*[4,5]). Multi-objective evolutionary algorithms (MOEA) lend themselves especially well to this application, as they are capable of generating or tuning neuronal models by optimizing multiple, often conflicting, objectives at the same time (*e.g.*[6,7]). The one weakness of MOEA-based construction of neuronal models, however, is the fact that it solely relies on the convergence efficiency of the chosen algorithm, and it largely ignores the plethora of available biological knowledge (other than what is utilized in the initial stages of model building in terms of the overall design of the model structure and the definition of fitness functions) that could be used to make the process itself more efficient. In this work, we propose to remedy this situation by hybridizing multi-objective evolutionary algorithms with a fuzzy logic-based controller in order to supply the MOEA with expert knowledge that can improve the algorithm's effectiveness. Fuzzy logic (FL) is a form of multiple-valued logic, which can emulate and incorporate human-like intelligence into a system controlling a process within a closed loop [8]. Fuzzy IF-THEN rules can be used to determine the best possible adjustment to the process based on its current output. Here, the process is the evolutionary algorithm, and the models generated by it are subjected to a set of fuzzy rules to determine the best adjustments, if any, to increase the likelihood of generating models that meet the predefined criteria. The application of fuzzy logic allows for utilization of linguistic rules that are easy to articulate and understand by humans (*e.g.* "IF spiking frequency is too low, THEN increase axon sodium conductance"), but at the same time can be directly applied to the neuronal models generated by the MOEA via the process of fuzzification (*i.e.* translation of crisp numerical values into linguistic concepts) and defuzzification, after the pertinent rules have been triggered. Importantly, as the loop continues to execute, more rules can be extracted from the evolutionary algorithm itself by simply identifying the changes across the generations that produced improvement, and mapping that information onto the fuzzy logic domain.

## References

[B1] Van Geit W De Schutter E Achard P Automated neuron model optimization techniques: a review Biol Cybern 2008 99 241 251 10.1007/s00422-008-0257-6 19011918

[B2] Prinz AA Billimoria CP Marder E Alternative to hand-tuning conductance-based models: Construction and analysis of databases of model neurons J Neurophysiol 2003 90 3998 4015 10.1152/jn.00641.2003 12944532

[B3] Hendrickson EB Edgerton JR Jaeger D The use of automated parameter searches to improve ion channel kinetics for neural modeling J Computat Neurosci 2011 31 2 329 346 10.1007/s10827-010-0312-x 21243419

[B4] Ben-Shalom R Aviv A Razon B Korngreen A Optimizing ion channel models using a parallel genetic algorithm on graphical processors J Neurosci Meth 2012 206 2 183 194 10.1016/j.jneumeth.2012.02.024 22407006

[B5] Smolinski TG Soto-Treviño C Rabbah P Nadim F Prinz AA Analysis of biological neurons via modeling and rule mining Int J Inf Tech and Intell Comp 2006 1 293 302

[B6] Druckmann S Banitt Y Gideon A Schurmann F Markram H Segev I A novel multiple objective optimization framework for automated constraining of conductance-based neuron models by noisy experimental data Front Neurosci 2007 1 7 18 10.3389/neuro.01.1.1.001.2007 18982116 PMC2570085

[B7] Smolinski TG Prinz AA Computational intelligence in modeling of biological neurons: A case study of an invertebrate pacemaker neuron Proc IJCNN 2009 2964 2970

[B8] Zadeh LA Fuzzy logic Scholarpedia 3 3 1766

